# Polymorphisms in the *CYP1B1* gene are associated with increased risk of prostate cancer

**DOI:** 10.1038/sj.bjc.6601288

**Published:** 2003-10-14

**Authors:** B L Chang, S L Zheng, S D Isaacs, A Turner, G A Hawkins, K E Wiley, E R Bleecker, P C Walsh, D A Meyers, W B Isaacs, J Xu

**Affiliations:** 1 Center for Human Genomics, Wake Forest University School of Medicine, Medical Center Boulevard, Winston-Salem, NC 27157, USA; 2 Department of Urology, Johns Hopkins Medical Institutions, Baltimore, MD, USA

**Keywords:** prostate cancer, association, hereditary, haplotype, *CYP1B1*

## Abstract

*CYP1B1* has been evaluated as a candidate gene for various cancers because of its function in activating environmental procarcinogens and catalysing the conversion of oestrogens to genotoxic catechol oestrogens. To test the hypothesis that genetic polymorphisms in the *CYP1B1* gene may associate with the risk for prostate cancer (CaP), we compared the allele, genotype, and haplotype frequencies of 13 single nucleotide polymorphisms (SNPs) of *CYP1B1* among 159 hereditary prostate cancer (HPC) probands, 245 sporadic CaP cases, and 222 unaffected men. When each of the SNPs was analysed separately, marginally significant differences were observed for allele frequencies between sporadic cases and controls for three consecutive SNPs (−1001C/T, −263G/A, and −13C/T, *P*=0.04–0.07). Similarly, marginally significant differences between sporadic cases and controls in the frequency of variant allele carriers were observed for five consecutive SNPs (−1001C/T, −263G/A, −13C/T, +142C/G, and +355G/T, *P*=0.02–0.08). Interestingly, when the combination of these five SNPs was analysed using a haplotype approach, a larger difference was found (*P*=0.009). One frequent haplotype (C-G-C-C-G of −1001C/T, −263G/A, −13C/T, +142C/G, and +355G/T) was associated with an increased risk for CaP, while the other frequent haplotype (T-A-T-G-T) was associated with a decreased risk for CaP. These findings suggest that genetic polymorphisms in *CYP1B1* may modify the risk for CaP.

The *CYP1B1* gene encodes an extrahepatic cytochrome *P*450 enzyme that activates many structurally diverse environmental procarcinogens, including polycyclic aromatic hydrocarbons (PAHs), heterocyclic and aryl amines, and nitroaromatic hydrocarbons (Shimada *et al*, 1996; Kim *et al*, 1998). When activated, these procarcinogens produce reactive intermediates that can cause DNA damage in cells. The importance of *CYP1B1* in chemical carcinogenesis has been demonstrated in a knockout mouse model (Buters *et al*, 1999; Heidel *et al*, 2000). Although *CYP1B1*-null mice were found to be resistant to 7,12-DMBA, among wild-type counterparts this known procarcinogen induced malignant lymphoma, as well as bone marrow cytotoxicity and preleukaemia. In addition to its role in procarcinogen activation, *CYP1B1* is also involved in the oxidative metabolism of oestrogens, as it preferentially catalyses the hydroxylation of oestrogens at the C-4 position to 4-hydroxy CEs (Hayes *et al*, 1996). 4-hydroxy CEs can form depurinated DNA adducts and was found to be carcinogenic in several animal models (Yager and Liehr, 1996). Owing to its role in the metabolism of both environmental and endogenous procarcinogens, *CYP1B1* is hypothesised to play an important role in carcinogenesis.

The human *CYP1B1* gene has been mapped to chromosomal region 2p21–22 (Sutter *et al*, 1994). *CYP1B1* consists of three exons, with the coding region starting in exon 2 (Tang *et al*, 1996). Many single nucleotide polymorphisms (SNPs) in the *CYP1B1* gene have been reported (http://www.ncbi.nlm.nih.gov/SN P/snp_ref.cgi?locusId=1545), of which four cause amino-acid substitutions (SNP *C142G*, *G355T*, *C4326G*, and *A4390G*, which result in Arg48Gly (R48G), Ala119Ser (A119S), Leu432Val (L432V), and Asn453Ser (N453S) amino-acid substitutions, respectively). The R48G substitution is located only two amino acids upstream of a highly conserved PPGP region, which is important for proper protein folding and stability (Nelson and Strobel, 1988; Johansson *et al*, 1994). The A119S substitution is located in substrate recognition site one (SRS1) (Gotoh, 1992), and may affect substrate binding. The other two nonsynonymous changes, L432V and N453S, are both located in exon 3, which encodes the haem-binding domain. Multiple functional studies report that these nonsynonymous SNPs of *CYP1B1* alter enzymatic activity and catalytic specificity. However, the results have not been consistent and are difficult to compare because different variants were tested (a total of 16 combinations for four amino-acid substitutions), and different expression and assay systems were used, in each laboratory (Hanna *et al*, 2000; Li *et al*, 2000; McLellan *et al*, 2000; Shimada *et al*, 2001; Aklillu *et al*, 2002).

The association between the polymorphisms of *CYP1B1*, especially L432V, and susceptibility to several cancers has been investigated. This includes smoking-related head and neck squamous cell cancer, colorectal, breast, ovarian, and prostate cancers (Bailey *et al*, 1998; Fritsche *et al*, 1999; Goodman *et al*, 2001; Ko *et al*, 2001; De Vivo *et al*, 2002; Tanaka *et al*, 2002; Tang *et al*, 2000; Watanabe *et al*, 2000; Zheng *et al*, 2000). However, few studies have thoroughly investigated the association of multiple SNPs in *CYP1B1* and cancer risk. Considering that sequence variants in the noncoding region may also affect the regulation and function of genes and the difficulty of functional characterisation of various *CYP1B1* isoforms *in vitro*, analysing multiple SNPs simultaneously may provide more direct evidence for the relationship between cancer risk and the genetic polymorphisms in *CYP1B1*. Based on the function of *CYP1B1* in activating procarcinogens and transforming oestrogens to genotoxic 4-hydroxyl-CEs, we hypothesise that polymorphisms in *CYP1B1* may affect the risk for prostate cancer. In addition, we hypothesise that polymorphisms in *CYP1B1* impose a different risk for hereditary prostate cancer compared to sporadic prostate cancer. To test these hypotheses, we estimated the frequencies and tested for differences in the frequencies of *CYP1B1* SNPs among 159 HPC probands, 245 sporadic prostate cancer cases, and 211 unaffected men.

## MATERIALS AND METHODS

### Subjects

HPC families (*n*=159) were recruited at the Brady Urology Institute at Johns Hopkins Hospital (Baltimore, MD, USA), through referrals, review of medical records for patients seen at Johns Hopkins Hospital for treatment of prostate cancer, and respondents to various lay publications describing our studies. The eligibility criterion for HPC was at least three first-degree relatives affected with prostate cancer. The diagnosis of CaP was verified by medical records for each affected male studied. The age of diagnosis of CaP was confirmed either through medical records or from two other independent sources. The mean age at diagnosis was 61 years; 134 (84%) were Caucasian and 14 (8.8%) were African American. The average number of affected men per family was 5.08.

Sporadic CaP cases (*n*=245) were from patients who underwent treatment for CaP at the John Hopkins Hospital and agreed to participate in the prostate cancer genetic study. Patients who met the criterion for HPC were excluded from this group. The diagnosis of CaP for all these subjects was confirmed by pathology reports. The mean age at diagnosis for these cases was 58.7 years; 229 (93%) were Caucasian and 8 (3.3%) were African American.

Non-CaP controls (*n*=222) were selected from men participating in screening programmes for CaP. By excluding subjects with abnormal PSA levels (i.e., ⩾4 ng ml^−1^), 217 were eligible for the study. The mean age at examination was 58 years; 188 (86.6%) of the eligible controls were Caucasian and 15 (6.9%) were African American. Based on interviews with the controls, about 5.5% of the eligible controls had brothers or a father affected with CaP.

All individuals who participated in this study gave full informed consent.

### Genotyping and statistical methods

In all, 20 SNPs distributed throughout the *CYP1B1* gene, including the promoter region, all exons, and all introns, were selected from Entrez dbSNP (http://www.ncbi.nlm.nih.gov/SN P/snp_ref.cgi?locusId=1545) and were then genotyped in 24 unrelated Caucasians to estimate the frequency, using the MassARRAY system (SEQUENOM Inc., San Diego, CA, USA) according to the manufacturer's recommendation. Among these 20 SNPs, we performed additional genotyping for 13 SNPs that were informative and frequent (frequency >5%) among all HPC probands, sporadic prostate cancer cases, and unaffected controls. As detailed in Table 1

**Table 1:** Pairwise linkage disequilibrium (correlation coefficient) between CYP1B1 polymorphisms in Caucasian control subjects

**Correlation coefficient/*P*-value**	**−1549A/G**	**−1001C/T**	**−263G/A**	**−13C/T**	**+142C/G (R48G)**	**+355G/T (A119S)**	**+3653C/A**	**+4326C/G (L432V)**	**+4379C/T (D449D)**	**+4390A/G (N453S)**	**+5359T/G**	**+5639G/A**	**+7072A/T**
−1549A/G		0.34	0.34	0.34	0.34	0.34	0.25	0.57	0.56	0.21	0.27	0.56	0.95
−1001C/T	0.000		1.00	1.00	1.00	0.99	0.33	0.60	0.60	0.28	0.36	0.62	0.35
−263G/A	0.000	0.000		1.00	1.00	0.99	0.32	0.60	0.60	0.28	0.36	0.62	0.36
−13C/T	0.000	0.000	0.000		1.00	0.99	0.32	0.60	0.60	0.28	0.36	0.62	0.36
+142C/G (R48G)	0.000	0.000	0.000	0.000		0.99	0.32	0.60	0.61	0.28	0.36	0.62	0.36
+355G/T (A119S)	0.000	0.000	0.000	0.000	0.000		0.32	0.61	0.60	0.29	0.36	0.62	0.36
+3653C/A	0.018	0.000	0.000	0.000	0.000	0.000		0.53	0.53	0.21	0.88	0.53	0.25
+4326C/G (L432V)	0.000	0.000	0.000	0.000	0.000	0.000	0.000		1.00	0.37	0.60	0.99	0.58
+4379C/T (D449D)	0.000	0.000	0.000	0.000	0.000	0.000	0.000	0.000		0.38	0.60	0.99	0.56
+4390A/G (N453S)	0.006	0.007	0.001	0.004	0.006	0.002	0.323	0.000	0.000		0.22	0.37	0.22
+5359T/G	0.006	0.000	0.000	0.000	0.000	0.000	0.000	0.000	0.000	0.143		0.60	0.28
+5639G/A	0.000	0.000	0.000	0.000	0.000	0.000	0.000	0.000	0.000	0.000	0.000		0.58
+7072A/T	0.000	0.000	0.000	0.000	0.000	0.000	0.019	0.000	0.000	0.003	0.001	0.000	

Note: The upper right cells are the estimates of pairwise correlation coefficient. The lower left cells are the *P*-values of pairwise LD tests.

, this sub-sample of 13 SNPs included two SNPs in the promoter region, four nonsynonymous and one synonymous SNPs in the coding region, two SNPs in intron1, 1 SNP in intron 2, and two SNPs in the 5′UTR region.

We performed both a Hardy–Weinberg equilibrium (HWE) test for each sequence variant and a pairwise linkage disequilibrium (LD) test for all sequence variants using the Fisher probability test statistic, as described by Weir (1996). For each test, 10 000 permutations were performed and the test statistic of each replicate was calculated. Empirical *P*-values for each test were estimated as the proportion of replicates that is as probable or less probable than the observed data, as implemented in the software package Genetic Data Analysis (GDA).

Tests for SNP allele frequency differences between cases and controls were performed using the *χ*^2^ with a degree of freedom of 1. An unconditional logistic regression was used to test for an association between genotypes and prostate cancer, after combining two types of variant allele carriers (heterozygotes or homozygotes) into one group and adjusting for age.

The haplotype frequency of unrelated individuals was estimated using the new statistical method proposed by Stephens *et al* (2001), as implemented in the computer program PHASE (http://www.stats.ox.ac.uk/math gen/software.html). Several runs using different values for the seed of the random number generator were performed and the goodness-of-fit values were similar among the different runs. An association between the haplotypes and prostate cancer risk was obtained using a score test developed by Schaid *et al* (2002), as implemented in the computer program HAPLO.SCORE (http://www.mayo.edu/statgen for the S-PLUS programming language or http://www.wfubmc.edu/docs/g enomics for the R programming language).

## RESULTS

Since over 90% of the study subjects were Caucasians, all analyses were limited to Caucasians only, to decrease the potential for population stratification. All the SNPs were in HWE (*P*>0.05) in each subset and were in strong LD, with the estimates of pairwise correlation coefficients ranging from 0.22 to 1.00 (Table 1). When each SNP was evaluated individually, marginally significant differences of allele frequencies between sporadic cases and controls were observed for three consecutive SNPs (−1001C/T, −263G/A, and −13C/T) (Table 2

**Table 2:** Allele frequencies of CYP1B1 polymorphisms

			**Controls**	**Sporadics**		**HPC probands**	
**SNP^a^**	**Location**	**Variant allele**	**No. of chromosomes with variant (no. of total chromosomes)**	**No. of chromosomes with variant (no. of total chromosomes)**	***P*-value^b^**	**No. of chromosomes with variant (no. of total chromosomes)**	***P*-value^c^**
−1549A/G	Promoter	G	70 (350)	98 (438)	0.42	46 (240)	0.9
−1001C/T	Promoter	T	109 (352)	110 (440)	0.07	71 (240)	0.63
−263G/A	Intron 1	A	112 (366)	111 (456)	0.05	74 (244)	0.87
−13C/T	Intron 1	T	112 (366)	113 (454)	0.07	71 (242)	0.66
+142C/G (R48G)	Exon 2	G	114 (368)	116 (452)	0.1	70 (238)	0.6
+355G/T (A119S)	Exon 2	T	115 (370)	121 (454)	0.16	74 (244)	0.77
+3653C/A	Intron 2	A	68 (366)	73 (446)	0.39	33 (232)	0.16
+4326C/G (L432V)	Exon 3	G	160 (364)	187 (414)	0.72	76 (206)	0.1
+4379C/T (D449D)	Exon 3	T	166 (370)	193 (424)	0.84	86 (226)	0.12
+4390A/G (N453S)	Exon 3	G	56 (364)	78 (404)	0.16	43 (224)	0.23
+5359T/G	3′UTR	G	74 (346)	87 (402)	0.92	48 (258)	0.34
+5639G/A	3′UTR	A	154 (346)	193 (428)	0.86	88 (236)	0.09
+7072A/T	3′UTR	T	77 (360)	107 (452)	0.42	48 (238)	0.88

a Numeric values represent the position (in base pairs) from the transcription start site. The letter represents nucleotide change. The letter and number in each parenthesis represent the amino acid change and the position of the amino-acid change.

b *P*-values of χ^2^ test for allele frequencies with df=1.

). Similarly, marginally significant differences of genotype frequencies between sporadic cases and controls were found for five consecutive SNPs (−1001C/T, −263G/A, −13C/T, +142C/G, and +355G/T (Table 3

**Table 3:** Genotype frequencies of CYP1B1 polymorphisms

		**Controls**	**Sporadics**		**HPC probands**	
**SNP**	**Genotypes**	**No. of subjects**	**No. of subjects**	***P*-value^a^**	**No. of subjects**	***P*-value^b^**
−1549A/G	AA	115	130		77	
	AG&GG	60	89	0.2	43	0.78
−1001C/T	CC	83	127		61	
	TC&TT	93	93	0.04	59	0.54
−263G/A	GG	87	132		62	
	AG&AA	96	95	0.03	59	0.53
−13C/T	CC	87	134		61	
	TC&TT	96	94	0.02	61	0.67
+142C/G (R48G)	CC	86	129		61	
	CG&GG	98	97	0.04	58	0.44
+355G/T (A119S)	GG	86	125		61	
	GT&TT	99	102	0.08	61	0.55
+3653C/A	CC	117	156		84	
	CA&AA	66	67	0.2	32	0.13
+4326C/G (L432V)	CC	53	55		39	
	CG&GG	129	152	0.58	64	0.13
+4379C/T (D449D)	CC	53	57		42	
	CT&TT	132	155	0.7	71	0.13
+4390A/G (N453S)	AA	132	131		72	
	AG&GG	50	71	0.11	40	0.14
+5359T/G	TT	102	124		84	
	TG&GG	71	77	0.59	45	0.28
+5639G/A	GG	50	58		45	
	GA&AA	123	156	0.7	73	0.1
+7072A/T	AA	112	127		74	
	AT&TT	68	99	0.22	45	0.99

a *P*- values of logistic regression adjusted for age.

b NS=not significant.

). For example, compared with the unaffected men (52.46%), the frequency of ‘T’ allele carriers of the SNP −13C/T was significantly lower in sporadic cases (41.23%, *P*=0.02). As these five SNPs were in strong LD (pairwise correlation coefficient ranging from 0.99 to 1.0), it is difficult to examine whether these observed associations were independent or an outcome of LD with a causal SNP. Interestingly, when the combination of these five SNPs was analysed using haplotype approaches, the haplotype frequencies were significantly different between sporadic cases and controls (Table 4

**Table 4:** *CYP1B1* haplotype frequencies

	**Frequencies**			**Score test (*P*-values)**	
**Haplotype**	**Controls**	**HPC**	**Sporadic**	**HPC *vs* controls**	**Sporadic *vs* controls**
(−1001C/T)-(−263G/A)-(−13C/T)-(+142C/G)-(+355G/T)					
C-G-C-C-G	0.683	0.68	0.731	0.39 (0.69)	1.05 (0.29)
T-A-T-G-T	0.312	0.316	0.242	−0.42 (0.67)	−1.9 (0.057)
Global				0.23 (0.845)	9.69 (0.009)
					
(+142C/G)-(+355G/T)- (+4326C/G)-(+4390A/G)					
G-T-C-A	0.31	0.32	0.24	−0.01 (0.93)	−1.75 (0.071)
C-G-G-A	0.44	0.38	0.43	−1.80 (0.07)	−0.24 (0.812)
C-G-C-A	0.09	0.13	0.13	1.17 (0.264)	0.52 (0.621)
C-G-C-G	0.15	0.17	0.16	1.34 (0.202)	0.17 (0.285)
Global				5.26 (0.263)	11.00 (0.02)

). Within the eight observed haplotypes, only two haplotypes, C-G-C-C-G and T-A-T-G-T (for SNPs −1001C/T, −263G/A, −13C/T, +142C/G, and +355G/T), were common. The remaining six haplotypes were observed less than 0.5% among our study population and were therefore excluded in the later analyses. A global haplo score test provided a significant difference between sporadic cases and controls, with a *P*-value of 0.009 (Table 4). The haplotype C-G-C-C-G was associated with increased risk for CaP, with a frequency of 73% in sporadic cases and 68% in controls. The other haplotype T-A-T-G-T was associated with a decreased risk for CaP, with a frequency of 24% in sporadic cases and 31% in controls. However, no significant difference was observed for allele, genotype, and haplotype frequencies between HPC probands and controls (Tables 2, 3 and 4).

Since most functional studies have evaluated the impact of four nonsynonymous changes on *CYP1B1* enzymatic activity, we also compared the haplotype frequencies of these four nonsynonymous changes: +142C/G (R48G), +355G/T (A119L), +4326C/G (L432V), and +4390A/G (N453S) between the cases and controls. Ten out of 16 possible haplotypes were observed in our study subjects. However, except for haplotypes G-T-C-A, C-G-G-A, C-G-C-A, and C-G-C-G, the rest of the haplotypes were rare (<0.5%) and not included in later analyses. Again, a significant difference between sporadic cases and controls was observed in a global haplo score test, with a *P*-value of 0.02 (Table 4). When specific haplotypes were examined, haplotype G-T-C-A was associated with a decreased risk for CaP, with the frequency of 24% in sporadic cases and 31% in controls. The frequencies of the remaining haplotypes were very similar between sporadic cases and controls. There was no significant difference in the haplotype frequency between HPC probands and controls.

## DISCUSSION

In this study, we tested for an association between polymorphisms in the *CYP1B1*gene and prostate cancer risk by comparing the allele, genotype, and haplotype frequencies of *CYP1B1* SNPs among HPC probands, sporadic prostate cancer cases, and unaffected men. Although no significant difference in the distributions of these SNPs between HPC probands and unaffected controls was found, differences in allele and genotype frequency between sporadic cases and controls were observed for several consecutive SNPs. Furthermore, haplotype analysis revealed larger differences between sporadic cases and unaffected controls and the diversity of haplotypes was limited in Caucasians. The haplotype C-G-C-C-G (for consecutive SNPs −1001C/T, −263G/A, −13C/T, +142C/G, and +355G/T) was found to be associated with an increased risk for sporadic prostate cancer, while the haplotype T-A-T-G-T was associated with a decreased risk for sporadic prostate cancer. Although the results cannot differentiate the contribution of specific SNPs to the observed association, they suggest that a specific segment of the gene is associated with prostate cancer risk. This is the first study that systematically evaluates the influence of multiple genetic variants in the *CYP1B1* gene on prostate cancer risk using a haplotype approach. It is also the first report to include hereditary prostate cancer patients, which enabled us to test whether the degree of association between the polymorphisms of *CYP1B1* and prostate cancer risk is strong enough to contribute to familial aggregation of prostate cancer.

Caution should be exercised when interpreting our findings. While the significant differences seen in the genotype and haplotype frequencies between prostate cancer cases and controls could be due to differential risks for prostate cancer caused by these polymorphisms, it could also be due to other reasons, such as a type I error or population stratification. All the reported significance levels were nominal *P*-values, and were not adjusted for multiple comparisons. When considering the fact that as many as 13 SNPs were tested in this report (for prostate cancer risk), if we use the commonly suggested Bonferoni correction, the results are not significant. However, the Bonferoni correction is not optimal in this case because not all of these tests were independent, due to LD between these polymorphisms and the dependence between allele, genotype, and haplotype. As a case–control study, the results are subject to potential population stratification: that is, the different genotype frequencies observed may partially reflect different genetic backgrounds in cases and controls. However, we feel that population stratification is unlikely to be substantial in this population because: (1) the statistical tests were limited to Caucasian subjects only, and (2) we observed no evidence for a significant difference in the genetic background between cases and controls, based on a sample of 24 consecutive SNPs recently genotyped on chromosomes 1, 8, 11, 12, and X (data not shown).

Two possible interpretations can be made for the observed larger difference between sporadic cases and unaffected men from the haplotype analysis. Firstly, the different haplotype frequencies may suggest a founder effect, that is, a substantial proportion of cases inherited the chromosomal segment at *CYP1B1* from a common ancestor. Secondly, because each of these variants could affect the function of *CYP1B1*, the specific combination of the variants on the same chromosome (in *cis* position) may have a particular phenotypic effect. Several laboratories have reported functional studies on the effects of *CYP1B1* polymorphisms on procarcinogens and oestrogen hydroxylation activities. Different allelic variants of *CYP1B1* have different catalytic activities and specificities to a variety of procarcinogens (Shimada *et al*, 2001). No specific allelic variants showed higher activities for every procarcinogen tested. In addition, due to different variant tests reported in each paper (a total of 16 combinations for four amino-acid substitutions) and different expression and assay systems used between different laboratories, the results were not consistent and were difficult to compare (Hanna *et al*, 2000; Li *et al*, 2000; McLellan *et al*, 2000; Shimada *et al*, 2001; Aklillu *et al*, 2002). It is difficult to draw conclusions from these previously reported studies and even more difficult to predict the impact of these variants on the risk of prostate cancer. In addition, the association between haplotypes and prostate cancer risk observed in our study also included polymorphisms in the intronic region immediately preceding exon 1 (−1001C/T, −263G/A, −13C/T). These intronic polymorphisms, either individually or in combination, may affect gene expression or mRNA splicing efficiency (Herrington *et al*, 2002). Further studies are needed to confirm these findings and to examine the mechanisms.

The studies between genetic polymorphisms in the *CYP1B1* gene and risk to a variety of cancers have focused on amino acid substitutions in exon 3, namely L432V and N453S. An association has been reported for the L432V polymorphism and risk for ovarian cancer (Goodman *et al*, 2001) and smoking-related head and neck squamous cell cancer (Ko *et al*, 2001). For breast cancer, while two studies (Bailey *et al*, 1998; De Vivo *et al*, 2002) failed to detect an association between L432V polymorphism and breast cancer risk in the Caucasian population, Zheng *et al* (2000) reported that the V allele of L432V increased breast cancer risk in Chinese women. Recently, Tanaka *et al* (2002) reported an association study between *CYP1B1* polymorphisms and prostate cancer risk in a Japanese population that included 117 prostate cancer cases and 200 controls. They found an opposite trend in terms of association of the risk allele with prostate cancer. Within four nonsynonymous SNPs and an SNP in intron 1 (−13C/T) that they examined, only SNP +355G/T (A119S) was statistically different between cases and controls, with the T allele being the risk allele (32% in cases compared to 15% in controls). However, we did not observe significant differences between cases and controls when we analysed SNP +355G/T (A119S) individually (T allele frequency: 27% in sporadic cases, 31% in controls, and 30% in HPC probands). The discrepancy between our results and their studies may be due to different risks in different ethnic populations. However, the deviation from HWE in their control group makes their results difficult to interpret. Although there was no association between SNPs in exon 3 and prostate cancer risk in our study population, we observed an association between SNPs near or within exon 1. It would be interesting to use other study populations to test the association between prostate cancer risk and several SNPs implicated by our study, including −1001C/T, −263G/A, −13C/T, +142C/G (R48G), and +355G/T (A119S). This is particularly true for the haplotypes of these SNPs.

No association between SNPs in the *CYP1B1* gene and the risk to hereditary prostate cancer was observed in this study, even though our study provided preliminary evidence for an association between *CYP1B1* and sporadic prostate cancer risk. This result indicates that either *CYP1B1* gene has no effect, or only plays a very minor role in familial aggregation of CaP. In fact, the power to detect a risk genotype in our hereditary prostate cancer population is limited when the genotype confers a low risk. For example, the power to detect a risk genotype that confers a relative risk of 1.8 to hereditary prostate cancer is only 70%, when the risk genotype is present in 50% of the control population. In addition, it is very likely that major susceptibility genes have a stronger effect in the hereditary families, which could mask the influence of minor genes.

In this study, we comprehensively studied the genetic variants of *CYP1B1* and the risk to prostate cancer, providing preliminary evidence for an association between *CYP1B1* haplotype and the risk to prostate cancer. Further studies with a larger sample size are needed to confirm our results.
